# Analysis of four long non-coding RNAs for hepatocellular carcinoma screening and prognosis by the aid of machine learning techniques

**DOI:** 10.1038/s41598-024-80926-w

**Published:** 2024-11-28

**Authors:** Ahmed Samir, Amira Abdeldaim, Ammar Mohammed, Asmaa Ali, Mohamed Alorabi, Mariam M. Hussein, Yasser Mabrouk Bakr, Asmaa Mohamed Ibrahim, Ahmed Samir Abdelhafiz

**Affiliations:** 1 Department of biochemistry, Faculty of pharmacy, October University for Modern Sciences and Arts (MSA), Giza, Egypt; 2 Faculty of computer science, October University for Modern Sciences and Arts (MSA), Giza, Egypt; 3 Department of Computer Sciences, FGSSR, Cairo University, Giza, Egypt; 4 Department of Chest Diseases, Abbasia Chest Hospital, Ministry of Health and Population, Cairo, Egypt; 5 Department of Clinical Oncology, Faculty of Medicine, Ain Shams University, Cairo, Egypt; 6 Department of Medical Oncology, Shefaa Al Orman Oncology Hospital, Luxor, Egypt; 7 Cancer Biology Department, National Cancer Institute, Cairo University, Cairo, Egypt; 8 Department of Clinical Pathology, Shefaa Al Orman Oncology Hospital, Luxor, Egypt; 9 Department of Clinical Pathology, National Cancer Institute, Cairo University, Cairo, Egypt

**Keywords:** HCC, lncRNAs, Machine learning, Screening, Oncology, RNA

## Abstract

Hepatocellular carcinoma (HCC) represents a significant health burden in Egypt, largely attributable to the endemic prevalence of hepatitis B and C viruses. Early identification of HCC remains a challenge due to the lack of widespread screening among at-risk populations. The objective of this study was to assess the utility of machine learning in predicting HCC by analyzing the combined expression of lncRNAs and conventional laboratory biomarkers. Plasma levels of four lncRNAs (LINC00152, LINC00853, UCA1, and GAS5) were quantified in a cohort of 52 HCC patients and 30 age-matched controls. The individual diagnostic performance of each lncRNA was assessed using ROC curve analysis. Subsequently, a machine learning model was constructed using Python’s Scikit-learn platform to integrate these lncRNAs with additional clinical laboratory parameters for HCC diagnosis. Individual lncRNAs exhibited moderate diagnostic accuracy, with sensitivity and specificity ranging from 60 to 83% and 53–67%, respectively. In contrast, the machine learning model demonstrated superior performance, achieving 100% sensitivity and 97% specificity. Notably, a higher LINC00152 to GAS5 expression ratio significantly correlated with increased mortality risk. The integration of lncRNA biomarkers with conventional laboratory data within a machine learning framework demonstrates significant potential for developing a precise and cost-effective diagnostic tool for HCC. To enhance the model’s robustness and prognostic capabilities, future studies should incorporate larger cohorts and explore a wider array of lncRNAs.

**Supplementary Information:**

The online version contains supplementary material available at 10.1038/s41598-024-80926-w.

## Introduction

Hepatocellular carcinoma (HCC) ranks as the sixth most prevalent cancer worldwide and the fourth most common cause of cancer-related mortality. The disease is one of the most aggressive human malignancies^1–3^. In Egypt, HCC ranks as the fourth most common cancer and the leading cause of cancer-related death^4^. The incidence of diagnosed HCC cases has doubled within the past decade^5^. While enhanced screening efforts have contributed to this increase, the predominant factor driving this trend is the endemic prevalence of hepatitis C virus (HCV) infection, the major risk factor for HCC development in Egypt^6^.

Advancements in treatment were associated with improved survival rates for cirrhotic patients, who have a higher risk of developing HCC^4^. Egypt’s pioneering national HCV screening and treatment campaign, initiated in 2018, represents a global benchmark in combating the disease. Through the administration of direct-acting antiviral therapy to over 2 million individuals, the program has been instrumental in identifying numerous HCC cases during follow-up surveillance using ultrasound and Alpha fetoprotein (AFP) testing^6,7^. AFP is a well-established biomarker for HCC screening in patients with chronic hepatitis and is a key diagnostic criterion when levels surpass 400 ng/mL, excluding pregnancy-related elevations. Approximately two-thirds of HCC patients exhibit elevated AFP levels^8,9^.

The low survival rate of HCC can be attributed to two key factors. First, the disease often presents asymptomatically in its early stages, making early diagnosis challenging. Second, effective treatment options are limited when the cancer is diagnosed at later stages, particularly after metastasis (10). This aggressive progression is driven by the accumulation of genetic and epigenetic alterations, ultimately leading to cancer development and metastasis^11^.

Treatment options for hepatocellular carcinoma (HCC) are highly dependent on tumor staging and liver function, as structured by the updated Barcelona Clinic Liver Cancer classification system. Early-stage HCC is typically treated with curative options such as surgical resection, ablation, or transplantation. Advanced disease warrants systemic therapies. While Sorafenib historically constituted the primary treatment, the current first-line standard includes anti-PD-L1 combination therapies, either with anti-VEGF agents or anti-CTLA-4 substances^12^.

Long noncoding RNAs (lncRNAs) are a class of non-coding RNAs greater than 200 nucleotides in length. These biomarkers are key regulators in several physiological and pathological processes. LncRNAs show differential expression patterns across diverse cancers, affecting their growth and survival potential^13^. During normal growth and development, lncRNAs play essential roles in modulating immune responses and regeneration, maintaining the liver microenvironment. However, the persistent proliferative signals caused by dysregulated lncRNAs often lead to liver tumorigenesis. Aberrant transcriptional or processing events may result in the upregulation of oncogenic lncRNAs or the silencing of tumor-suppressing lncRNAs, leading to conditions such as chronic hepatitis, liver overgrowth, and oxidative stress, which in turn drive the initiation and progression of hepatocellular carcinoma (HCC)^14^.

In HCC, numerous lncRNAs have been studied and found to promote many of these hallmarks such as proliferation, invasion, angiogenesis, and migration, while inhibiting cellular apoptosis^15^. These functions are mediated through mechanisms such as binding to DNA, RNA, or proteins, inducing epigenetic modifications, encoding small peptides, or acting as miRNA sponges that affecting their activities^14^. Recently many studies investigated the role of several lncRNAs in HCC progression; for instance LINC00152 found to promote cell proliferation through the regulation of CCDN1^16^. UCA1 was found to have similar effect on the proliferation and apoptosis of HCC however the exact mechanism is not completely revealed yet^17^. HOTAIR was found to be associated with poor overall survival and disease-free survival in HCC patients. Other lncRNAs, such as H19 and MALAT1, have also been linked to HCC progression and poor prognosis. MALAT1 has been found to promote aggressive tumor phenotypes and facilitate progression^18^. On the other hand, some lncRNAs were found to have a role in the inhibition of cancer cells proliferation and activation of apoptosis such as GAS5 which act by triggering CHOP and caspase-9 signal pathways^19^.

HCC-associated lncRNAs are detectable in body fluids, making them accessible and analyzable, which highlights their potential as valuable biomarkers for liquid biopsy in HCC. Emerging studies indicate that the expression levels of specific lncRNAs in the bloodstream offer promise as non-invasive biomarkers for the early detection and management of HCC^15^. Previous studies have identified several lncRNAs, including ENSG00000258332.1, LINC00635, SNHG1, LINC00152, LINC00853, HULC, UCA1 and other lncRNAs as potential diagnostic markers^20–23^. For example, serum lncRNA-WRAP53 has been identified as an independent prognostic marker, capable of predicting a high relapse rate in HCC patients^24^. Another study has used lncRNA-WRAP53 in combination with UCA1 and AFP to improve the prediction power^20^. Similarly, LINC00152 has been reported as a potential biomarker for HCC diagnosis, the reports also reflected its better diagnostic power upon its combination with AFP or with both AFP and HULC^21,22^. These studies confirmed that, These lncRNAs represent promising candidates as early diagnostic biomarkers, enabling timely intervention and potentially enhancing patient outcomes, especially if a combination of multiple lncRNAs are used alongside with the well-defined HCC biomarkers such as AFP^25^.

This study aimed to evaluate the diagnostic and prognostic utility of four lncRNAs: UCA1, GAS5, LINC00152, and LINC00853; selected based on previous literature^20–23,26^ and to use them as a combined diagnostic panel in integration with conventional liver function biomarkers. We also developed a machine learning (ML) model for accurate diagnosis of HCC using a combination of laboratory data including plasma levels of the selected lncRNAs and standard laboratory liver function tests.

## Patients and methods

### Study population and sample collection

Fifty-two newly diagnosed adult patients with HCC were recruited from the Medical Oncology Department of Shefaa Al Orman Oncology Hospital, Egypt. Thirty Age-matched healthy controls were also included in the study, sample size was calculated targeting power of 80% and confidence level of 95%, means and standard deviation of the studied lncRNAs expression levels from previous studies were used for calculations. Plasma samples were obtained from both groups: for HCC patients, samples were retrieved from the Shefaa Al Orman Biobank (SOH-BB), while control samples were collected following standard protocols. All participants provided written informed consent for study participation. The study protocol was approved by the ethical committee of Shefaa Al Orman under reference number SOH-IRB 09/2023.

Eligible patients were adults 18 years or older diagnosed with HCC according to the LI-RADS imaging criteria or histopathological examination of tissue biopsy. All patients were treatment-naive before sample collection. The control group consisted of age- and gender-matched healthy individuals without a history of liver disease, cancer, or chronic inflammatory disorders. These individuals were selected from the pool of blood donors in Shefaa Al Orman hospital.

Exclusion criteria included patients on immunosuppressive drugs, a history of chronic inflammatory diseases, non-HCC liver tumors, or other past or concurrent malignancies. Additionally, patients were excluded in case of incomplete medical records or insufficient available samples. Patients younger than 18 years old, with conditions such as hereditary hemorrhagic telangiectasia, Budd-Chiari syndrome, or cirrhosis due to congenital hepatic fibrosis, were also excluded to avoid false-positive results.

## Clinical and laboratory data

Clinical and laboratory data were collected from the medical records of all HCC patients. This included measurements of serum levels for alanine aminotransferase (ALT), aspartate aminotransferase (AST), AFP, total bilirubin, and albumin. These same laboratory tests were also performed on the control plasma samples.

## RNA isolation and cDNA synthesis

Total RNA was isolated from samples using the miRNeasy Mini Kit (QIAGEN, cat no. 217004) according to the manufacturer’s protocol. Reverse transcription into complementary DNA (cDNA) was carried out using the RevertAid First Strand cDNA Synthesis Kit (Thermo Scientific, cat no. K1622). The reverse transcription reaction was performed on a T100 thermal cycler (Bio-Rad).

## Quantitative real-time PCR (qRT-PCR)

To quantify the relative expression levels of the four lncRNAs, qRT-PCR was employed. The PowerTrack SYBR Green Master Mix kit (Applied Biosystems, cat no. A46012) and a ViiA 7 real-time PCR system (Applied Biosystems, Foster City, CA, USA) were used for this purpose. Primer sequences for qRT-PCR, designed by Thermo Fisher Scientific, are provided in Table 1. The housekeeping gene glyceraldehyde-3-phosphate dehydrogenase (GAPDH) was used for normalization of expression data. Each qRT-PCR reaction was performed in triplicate. The ΔΔCT method was used for relative quantification and data analysis, with results expressed accordingly^27^.

**Table 1:** Sequences of used primers for qRT-PCR.

	Sense	Antisense
Linc00152	GACTGGATGGTCGCTTT	CCCAGGAACTGTGCTGTGAA
LincC00853	AAAGGCTAGGCGATCCCACA	ACTCCCTAGCTTGGCTCTCCT
UCA1	TGCACCGACCCGAAACT	CAAGTGTGACCAGGGACTGC
GAS5	TCCCAGCCTCAGACTCAACA	TCGTGTCCCCGGATACTTTG
GADPH	GGGAAACTGTGGCGTGAT	GAGTGGGTGTCGCTGTTGA

### Statistical analysis

Statistical analysis was performed using Minitab 17.1.0.0 for Windows (Minitab Inc., 2013). Data normality was assessed with the Shapiro-Wilk test. Continuous variables were presented as medians and interquartile ranges (IQR), while categorical variables were expressed as frequencies and percentages. Non-parametric data comparisons between patients and controls were performed using the Mann-Whitney U test for numerical data and Chi-square tests for categorical data. Receiver operating characteristic (ROC) curves were generated to evaluate the diagnostic potential of lncRNAs, AFP, ALT, and AST for HCC. A general linear model with stepwise forward selection was used to identify factors influencing lncRNA levels in HCC patients. Multiple logistic regression analysis, adjusted for age and sex, was employed to assess the role of lncRNAs in predicting HCC mortality. All statistical tests were two-sided, and a p-value of less than 0.05 was considered statistically significant.

### Machine learning model development

Machine learning models for diagnosis of HCC using a combination of laboratory data including lncRNAs and other laboratory data was implemented using Python libraries Scikit-learn. The models were implemented using different classification algorithms, such as Gaussian Naïve Bayes, Gradient Boosting, support vector machine and logistic regression, to compare their predictive performance and select the outperforming model. Our model choices were based on the following reasoning. SVM is well-suited for high-dimensional nonlinear separable datasets. Also, SVM’s ability to maximize the margin between classes (especially binary classes) makes it robust to overfitting, especially with smaller datasets. Naïve Bayes is an algorithm that provides a probabilistic framework that can be useful for medical diagnosis, where probabilistic outputs can aid clinical decision-making. Gradient Boosting is an ensemble method known for its ability to model complex, non-linear relationships by combining weak learners (usually decision trees). It performs well in cases where feature interactions are unknown or difficult to model explicitly. Finally, Logistic regression is a simple linear model that provides interpretable results.

Those machine learning models were experimented using different combinations of laboratory data. The steps to implement a machine learning model includes data selection, data cleaning and normalization, data transformation, data splitting to training set and validation set and finally model training using classification algorithm with evaluation using different metrics. We utilized cross validation techniques, 100 different splitting of data sets, in the training and validation to get robust predictive models.

To enhance the robustness of our predictive models and ensure accurate performance evaluation, we adopted the RepeatedStratifiedKFold cross-validation technique. Specifically, we used 5 folds (k = 5) and repeated the process 5 times. This method ensures that the class distribution is preserved in each fold, and the cross-validation process is repeated multiple times, each time with a different random split. By employing RepeatedStratifiedKFold with 5 folds repeated 5 times, we reduced the variance of the model evaluation metrics, providing a more stable estimate of model performance and preventing overfitting to a specific train-test split. This approach allowed each model, from the model to be trained and validated on 25 different data splits (5 folds × 5 repetitions), ensuring that the performance estimate was less likely to be overly optimistic or pessimistic, which can happen if only a single cross-validation is performed.

## Results

### Clinical and demographic characteristics of participants

The study population consisted of adult patients with a median age of 63 years (IQR: 58–68 years). Males comprised most participants (81.01%). Other clinical features, data about staging, and treatment regimens are presented in Table 2.

**Table 2:** Clinical features of HCC patients.

Factors	HCC (*n* = 58)		
	N	%	
HCV (positive)	40	68.97	
HBV (positive)	7	12.07	
Cirrhosis (Yes)	52	89.66	
DAA (Yes)	14	24.14	
Ascites (Yes)	10	17.24	
Portal vein thrombosis (Yes)	17	29.31	
	Median (Q2)	Q1	Q3
AST	60	39.3	99.8
ALT	43.5	31	76.3
Total bilirubin	1.25	0.8	2.48
Albumin	3.7	3	3.9
INR	1.22	1.16	1.29
AFP	225	23	5587
Tumor size	7.2	4.8	10
*BCLC staging*	N	%	
Zero	3	5.17	
A	8	13.79	
B	13	22.41	
C	7	12.07	
D	27	46.55	
TACE (Yes)	13	22.41	
Radiofrequency Ablation (Yes)	5	8.62	
Resection (Yes)	1	1.72	
Sorafenib (Yes)	7	12.07	
BSC (Yes)	29	50	
Mortality	47	81.03	

Numerical data presented as median (Q2) and (Q1-Q3) and categorical data as number and percentage, N: number, Q: quartile, HCC: hepatocellular carcinoma.

### lncRNA expression

**Fig. 1:**
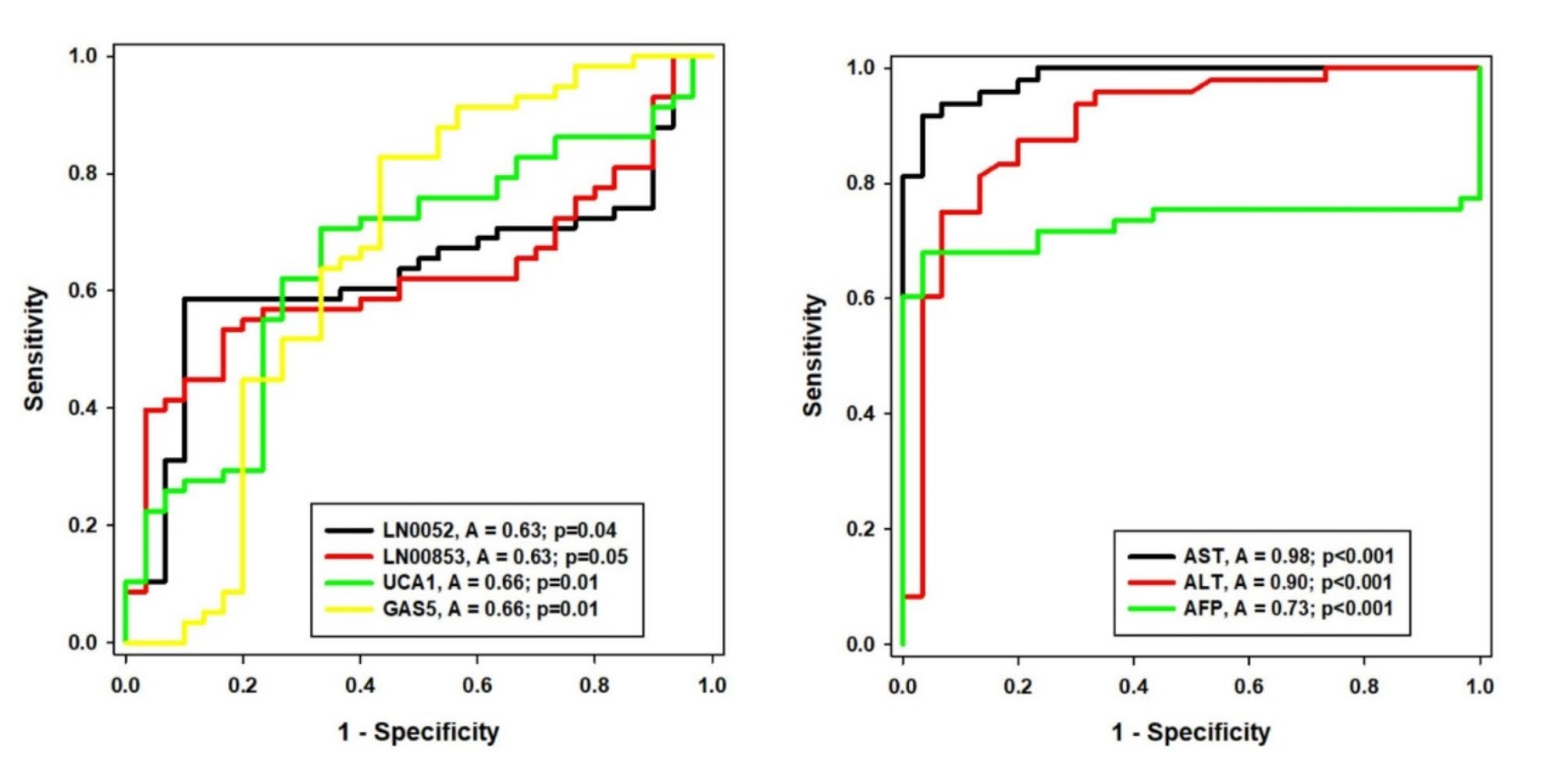
ROC curve of LN0052, LN00853, UCA1 and GAS5 long non-coding RNA and AST, ALT and AFP. A: area under curve, *p*< 0.05 considered significant.

Analysis of lncRNA expression showed significantly higher levels of LNC0052, LNC00853, UCA1, and GAS5 in HCC patients compared to controls (p-values = 0.04, 0.05, 0.01, and 0.01, respectively). Similarly, serum levels of AST, ALT, total bilirubin, and AFP were significantly elevated in HCC patients (p-value < 0.01 for all) (Table 3). ROC curve analysis (Fig. 1) demonstrated moderate power of discrimination for lncRNAs, with area under the curve (AUC) ranging from 63 to 66%. While AFP exhibited good discrimination (AUC = 73%). Comparative analysis of AUC values revealed AST as the superior marker compared to ALT and AFP, while no significant differences were observed among the lncRNAs (Supplementary Tables 1 and 2). While AST and ALT demonstrated strong discriminatory power, as evidenced by AUC values of 98% and 90% respectively, their lack of specificity for HCC renders them unsuitable as standalone diagnostic markers. The integration of these traditional liver function tests with lncRNA biomarkers is imperative to enhance diagnostic accuracy. Table 4 summarizes the optimal cut-off values, selected as the point with both highest sensitivity and specificity, for differentiating healthy controls from HCC patients. Sensitivity for the selected lncRNAs ranged from 60 to 83%, while specificity ranged from 53 to 67%.

**Table 3:** Expression of LN0052, LN00853, UCA1 and GAS5 long non-coding RNA in patients with HCC.

Factors	Control (*n* = 30)			HCC (*n* = 58)			*p*
	Median (Q2)	Q1	Q3	Median (Q2)	Q1	Q3	
Age	62	55	67	63	58	68	0.81^†^
*Sex*	N	%		N	%		
Male	23	76.6		47	81.03		0.15^*^
Female	7	23.4		11	18.97		
*LncRNA*	Median	Q2	Q3	Median	Q2	Q3	
LN0052	2.092	0.834	3.542	5.291	0.285	12.234	0.04^†^
LN00853	1.506	0.511	3.092	4.614	0.424	20.996	0.05^†^
UCA1	1.528	0.302	6.345	6.159	1.347	19.47	0.01^†^
GAS5	0.588	0.0545	11.896	7.8	0.9	15.966	0.01^†^
AST	9.05	6.10	16.38	60.00	39.25	99.75	0.001^†^
ALT	11.30	8.18	20.15	43.50	31.00	76.25	0.001^†^
Total bilirubin	110.05	77.67	144.93	1.25	0.80	2.48	0.001^†^
Albumin	3.55	3.13	3.90	3.70	3.00	3.90	0.77^†^
AFP	26.80	20.13	32.73	225.00	22.50	5586.50	< 0.001^†^

Numerical data presented as median (Q2) and (Q1-Q3) and categorical data as number and percentage, N: number, Q: quartile, HCC: hepatocellular carcinoma, The test of significant: †: Mann Whitney test, *: Chi square test, *p* ≤ 0.05 is considered significant.

**Table 4:** Diagnostic utility of LN0052, LN00853, UCA1 and GAS5 long non-coding RNA in HCC.

LncRNAs	Cutoff >	Sensitivity	95% CI	Specificity	95% CI	PPV	NPV
LN0052	2.78	60%	0.4664 to 0.7295	63%	0.4386 to 0.8007	27%	88%
LN00853	1.55	62%	0.4837 to 0.7449	53%	0.3433 to 0.7166	23%	87%
UCA1	1.99	71%	0.5727 to 0.8191	67%	0.4719 to 0.8271	32%	91%
GAS5	0.68	83%	0.7057 to 0.9141	57%	0.3743 to 0.7454	30%	94%
AST	23.70	94%	0.8280 to 0.9869	93%	0.7793 to 0.9918	93%	94%
ALT	24.05	83%	0.6978 to 0.9252	83%	0.6528 to 0.9436	83%	83%
AFP	32.8	72%	0.5765 to 0.8321	77%	0.5772 to 0.9007	75%	73%

CI: confidence interval, PPV: positive predictive value, NPV: negative predictive value.

### Associations between lncRNAs and clinical features

Table 5 summarizes the associations between lncRNA expression and various liver conditions in HCC patients. Positive Hepatitis C virus (HCV) infection correlated with elevated LINC00152 expression (*p* = 0.001) but decreased UCA1 expression (*p* = 0.05). Conversely, positive Hepatitis B virus (HBV) infection was only associated with increased LINC00853 expression (*p* = 0.001). Interestingly, liver cirrhosis displayed a distinct lncRNA profile: UCA1 upregulation and downregulation of LINC00152 and GAS5 (*p* = 0.02, 0.001, and 0.001, respectively).

**Table 5:** Factors influencing the expression of lncRNAs.

Factors	LN0052		LN00853		UCA1		GAS5	
	Coeff.	*P*	Coeff.	*P*	Coeff.	*P*	Coeff.	*P*
HCV (positive)	Reference		Reference		Reference		Reference	
Negative	-90.70	**0.001**	-30.90	0.08	3727.00	**0.05**	-23.70	0.12
HBV (positive)	Reference		Reference					
Negative	-58.40	0.08	-108.30	**0.001**				
Cirrhosis (Yes)	Reference				Reference		Reference	
No	118.70	**0.001**			-6612.00	**0.02**	69.90	**0.001**
*BCLC staging*								
Zero	Reference		Reference		Reference		Reference	
A	186.50	**0.001**	69.00	0.06	19295.00	**0.001**	216.40	**0.001**
B	-45.80	0.26	-43.30	0.14	-5418.00	0.11	-48.70	0.08
C	-32.90	0.53	55.50	0.15	-8563.00	**0.05**	-32.70	0.35
D	-60.10	0.09	-36.50	0.16	-4472.00	0.12	-66.80	**0.01**
Radiofrequency Ablation (Yes)	Reference				Reference		Reference	
No	97.90	**0.05**			9996.00	**0.02**	131.80	**0.001**

Coeff.: Coefficient, The test of significance: General linear model, with stepwise forward selection methods, *p* ≤ 0.05 considered significant, the sign before coeffeient denoting the direction of relationship.

### lncRNAs and mortality prediction

Our cohort exhibited a high mortality rate exceeding 81% regarding a one year time window. Table 6 shows the potential of lncRNAs as mortality predictors after adjusting for age and sex. Notably, higher levels of LINC00152 and lower levels of GAS5 were significantly associated with an increased risk of mortality (OR = 1.01 and 0.98 with *p* = 0.02 and 0.001, respectively).

**Table 6:** Role of lncRNAs expression in predicting mortality of HCC.

Factors	Coefficient	OR	95% CI	*p*
Age	-0.06	0.94	(0.8482,1.0428)	0.18
LN0052	0.01	1.01	(0.9754,1.0385)	**0.02**
LN00853	0.04	1.04	(0.9501,1.1323)	0.18
UCA1	0.001	1.00	(0.9997,1.0002)	0.53
GAS5	-0.02	0.98	(0.9596,1.0010)	**0.001**
M-Sex	-0.17	0.98	(0.9553,1.0144)	0.89

OR: odd ratio, CI: confidence interval, the test of fitness: Hosmer-Lemeshow, X^2^ = 3.4, *p* = 0.98, the test of significant: Multiple logistic regression model with adjustment for age and sex, *p* ≤ 0.05 considered significant.

### LncRNAs and survival probability

Following patient classification based on the lncRNA cutoff point in Table 4 the Kaplan-Meier analysis with log-rank tests revealed no significant difference in overall survival (defined as the interval between the initial diagnosis and death from any cause) probability between the higher and lower expression groups as shown in Table 7. Moreover, Cox regression analysis revealed no significant association between lncRNA expression and overall survival. (Supplementary Table 3).

**Table 7:** Survival time in different higher and lower expression groups of lncRNAs.

LncRNAs	*N*	Events (death)	Median Time (month)	*p*
*LN0052*				
> 2.78	35	29	9	0.95
< 2.78	23	18	6	
*LN00853*				
> 1.55	36	30	9	0.57
< 1.55	22	17	6	
*UCA1*				
> 1.99	41	34	9	0.69
< 1.99	17	13	6	
*GAS5*				
> 0.68	48	39	9	0.84
< 0.68	10	8	6	

The test of significance: Kaplan Meier with log-Rank test, *p* ≤ 0.05 considered significant.

### Diagnostic performance with machine learning

The machine learning model achieved the best prediction accuracy by combining traditional laboratory data (ALT, AST, total bilirubin, albumin, and AFP) with lncRNAs. This combined approach significantly improved prediction power compared to using traditional data alone or individual lncRNAs. Support Vector Machine (SVM) and Logistic Regression algorithms showed the strongest performance, reaching recall (sensitivity) of 100% and 97% with precision of 93% and 96.7%, respectively. Even after exclusion of ALT and AST from the model and including only lncRNAs, AFP, bilirubin, and albumin, the models achieved a sensitivity of 93% and precision of 97.5%. in contrast, removal of ALT and AST from the model using only traditional data, without lncRNAs, caused a noticeable decline in the prediction power. These results indicate superior importance of lncRNAs over ALT and AST despite the high differentiation power of ALT and AST between control and HCC. The superiority of lncRNAs is obviously due to their specific increase with HCC but not the other hepatic conditions in contrast with ALT, AST and the other traditional liver function markers. This data doesn’t deny the role of traditional liver function tests data for the model but actually reflects the importance of the integration of lncRNAs data with the other liver function data for more accurate and specific prediction of HCC, this aim is perfectly achieved by using the machine learning model with the whole data panel. Table 8 shows the results of AI models using different combinations of data sets.

**Table 8:** Results of machine learning models using different panels of data.

Data panel	Model	Accuracy	Precision	Sensitivity	F1_score
All data*	Support Vector Machine	0.959	0.95	0.967	0.951
	Logistic Regression	0.959	0.925	1	0.957
All without ALT and AST	Support Vector Machine	0.959	0.975	0.933	0.946
	Logistic Regression	0.959	0.975	0.933	0.946
Traditional data**	Support Vector Machine	0.95	0.975	0.9	0.926
	Logistic Regression	0.95	0.975	0.9	0.926
Traditional without ALT and AST	Support Vector Machine	0.898	0.885	0.9	0.872
	Logistic Regression	0.923	0.925	0.9	0.897

*all data panel include the 4 selected lncRNAs in addition to ALT, AST, Total bilirubin, albumin and AFB.

**traditional data paned includes only ALT, AST, Total bilirubin, albumin and AFB.

### Correlation matrix analysis

**Fig. 2:**
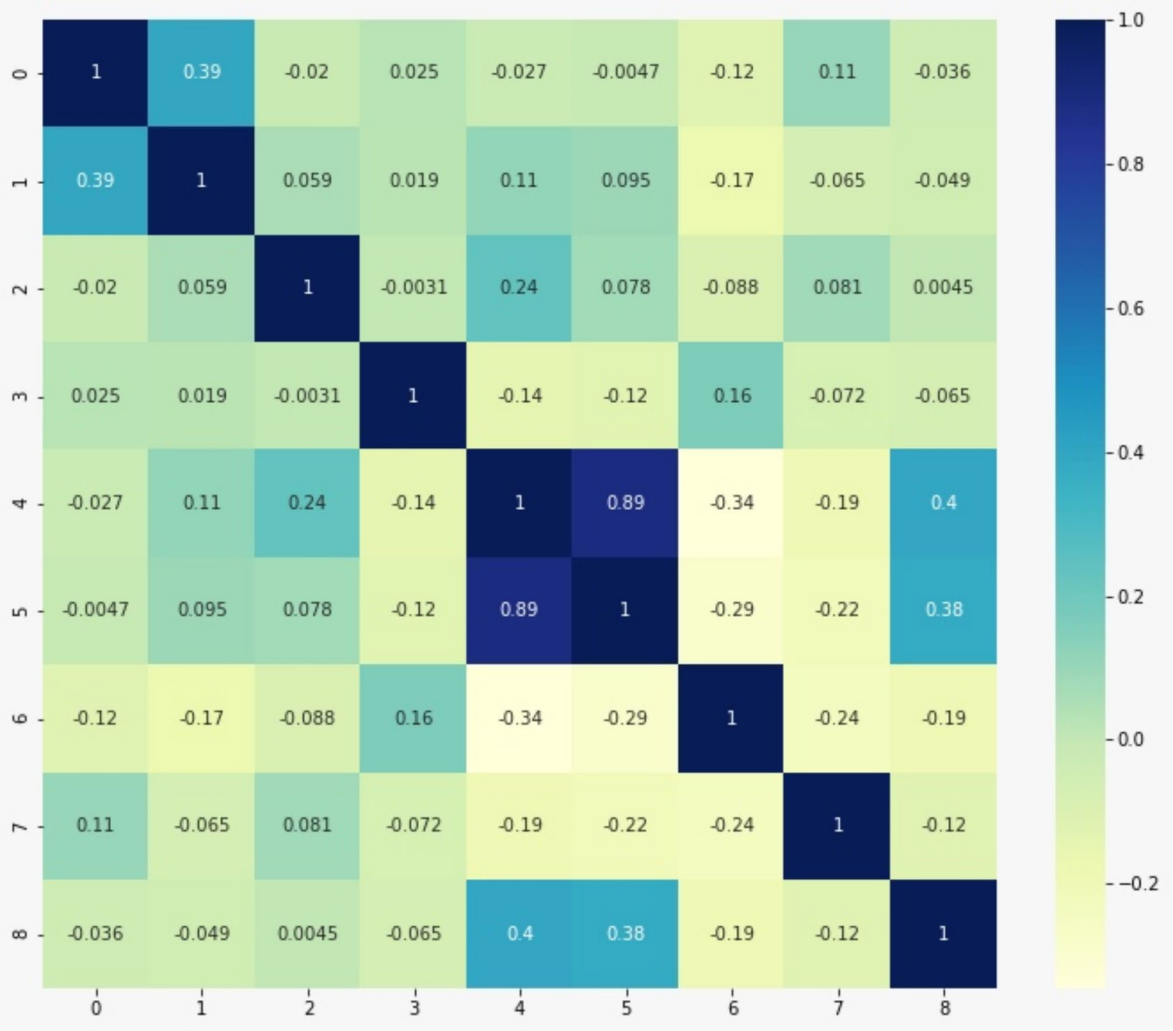
Correlation matrix shows the correlation between each pair of the biomarkers used in the study 0 = LINC00152, 1 = LINC00853, 2 = UCA1, 3 = GAS5, 4 = AST, 5 = ALT, 6 = total bilirubin, 7 = albumin and 8 = AFP.

Figure 2 presents the correlation matrix for all included laboratory data. The figure shows minimal to weak correlations between most studied factors, apart from ALT and AST, which exhibited a strong positive correlation as expected. These correlation matrix results support the importance of considering multiple parameters, including lncRNAs, for optimal model accuracy.

## Discussion

Hepatocellular carcinoma (HCC) poses a significant public health challenge in Egypt. Early detection is crucial for optimal patient outcomes. This study aimed to develop a machine learning model for improving HCC diagnosis by integrating long non-coding RNA (lncRNA) biomarkers with conventional liver function tests. In hepatocellular carcinoma (HCC), conventional biomarkers such as ALT and AST are commonly elevated due to liver damage, but this elevation is non-specific, occurring in various liver conditions, including hepatitis, cirrhosis, and general liver injury^9^. ALT and AST measure hepatocyte integrity but lack specificity to the molecular alterations unique to HCC. In contrast, lncRNAs like UCA1, GAS5, LINC00152, and LINC00853 are involved in HCC-specific oncogenic processes, including the regulation of cellular proliferation, apoptosis, and metastasis, which directly correlate with cancer pathology^13^. These lncRNAs provide insight into the molecular underpinnings of HCC that ALT and AST do not capture. By integrating these lncRNAs into our diagnostic model alongside ALT and AST, we significantly improved specificity and sensitivity, enhancing our ability to distinguish HCC from other liver conditions more accurately. This integration employs the unique predictive information offered by lncRNAs, which enhances the overall diagnostic power of the model and addresses the limitations of traditional liver enzymes in HCC screening.

Previous studies reported the changes in the expression of lncRNAs in HCC tissues^28^ and their elevated levels in patients’ serum samples^26^. All lncRNAs selected in this study have established roles in HCC pathogenesis, UCA1, GAS5, LINC00152, and LINC00853, were chosen based on their established functional relevance in hepatocellular carcinoma (HCC) and their demonstrated potential as diagnostic biomarkers. UCA1 is extensively documented as an oncogenic lncRNA involved in various malignancies, including HCC. It promotes cellular proliferation, migration, and resistance to apoptosis, partially through its interaction with key pathways such as the Hippo pathway, which influences tumor growth and survival^17^. Elevated UCA1 expression has been associated with poor outcomes in HCC patients, suggesting its potential as an indicator of aggressive disease progression^13^. On the other hand, GAS5 functions in contrast as a tumor suppressor, with its downregulation in HCC linked to enhanced proliferation and reduced apoptosis. Studies indicate that GAS5 plays a role in cell cycle arrest and apoptosis through mechanisms such as caspase-dependent endoplasmic reticulum stress pathways^11^. These contrasting roles of UCA1 and GAS5 provide complementary insights into the disease biology and justify their inclusion as diagnostic markers. LINC00152 has been shown to promote cell proliferation and migration through modulation of cyclin D1 (CCND1), with high expression linked to poor prognosis^16^. Furthermore, LINC00152 acts as a competing endogenous RNA (ceRNA), affecting oncogenic pathways by binding miRNAs that regulate tumor suppressor genes. LINC00853, although less extensively studied in HCC, has emerging evidence supporting its role in cellular proliferation and invasion, making it a promising candidate for further investigation as a diagnostic biomarker for HCC^23^. Although several lncRNAs were initially tested early during this study, our final model prioritized the combination of these selected four lncRNAs that optimized predictive accuracy while minimizing complexity and cost.

Our findings show that lncRNAs alone offer moderate sensitivity and specificity for HCC diagnosis. Additionally, some of the investigated lncRNAs demonstrated a prognostic association with mortality risk. The machine learning model we implemented significantly enhanced diagnostic sensitivity and specificity, which highlights the potential of this approach for improved early screening and diagnosis of HCC.

To our knowledge, this study represents a pioneering effort in utilizing a machine learning model for HCC diagnosis by integrating lncRNAs with standard laboratory data. Using the data processing capabilities of machine learning, we achieved significant improvement in diagnostic performance, with sensitivity and specificity approaching 100%. Furthermore, the developed model was translated into a user-friendly web application, which was piloted by healthcare professionals. Their feedback indicated a straightforward user interface that delivers rapid and accurate results based on laboratory data. This cost-effective approach holds promise for large-scale screening, enabling cost-efficient testing of a vast population compared to conventional diagnostic methods. Utilizing readily available laboratory data for screening has the potential to decrease the financial burden on the healthcare system, facilitating broader and more efficient service delivery.

Previous research has evaluated the use of artificial intelligence and accuracy of machine ML for prediction and/or diagnosis of HCC, and documented variations in the accuracy of different models. Sato et al. compared different algorithms (logistic regression, SVM, gradient boosting) using clinical data and found that gradient boosting exhibited the highest accuracy^29^. Angelis et al. who used a publicly available HCC dataset to evaluate different techniques for feature selection and classification, also achieved the best results (84% accuracy, 93% precision) with gradient boosting^30^. Wong et al. reported that ridge regression and random forest models offered comparable performance to traditional scores such as CU-HCC (California University-Hepatocellular Carcinoma) and GAG-HCC (Ghent-Amsterdam-Gothenburg-Hepatocellular Carcinoma) for HCC prediction in HBV/HCV patients^31^. In our study, Support Vector Machine and Logistic Regression algorithms showed the strongest performance. These findings highlight the importance of algorithm selection and potential variations in model performance for HCC diagnosis. On the other hand, although the performance of the developed model in this study approaches 100%, the results of machine learning model are somewhat sensitive to sample size, experimental setup and data sets. So the performance might be different with different data sets which make the validation of the model with different data sets highly recommended to ensure the clinical applicability.

Studies have also investigated the use of genetic data in ML models for HCC prediction. Chen et al. used a random forest model to investigate the potential of HBV reverse transcriptase gene potential HCC prediction. Their model achieved optimal performance using a combination of 10 features, demonstrating robustness across diverse HBV genotypes and sequencing depths^32^. Similarly, Tao et al. applied a random forest model to differentiate HCC from chronic HBV infection based on ctDNA copy number aberrations. The model achieved robust performance in the two validation cohorts they evaluated^33^.

Our study identified a significant association between increased mortality risk in HCC patients and both higher expression levels of LINC00152 and lower expression levels of GAS5. LINC00152, is known to be aberrantly expressed in various cancers, and has been linked to cell proliferation, migration, invasion, therapeutic resistance, tumor growth and metastasis^34^. Previous research established LINC00152 overexpression in HCC tissues compared to healthy controls and demonstrated its role as an independent prognostic factor associated with poorer patient survival^35,36^, suggesting its potential as a therapeutic target for HCC^37^.

In contrast to LINC00152, GAS5 demonstrated a protective effect against mortality in HCC patients, despite exhibiting higher expression levels in HCC compared to controls. Prior studies have documented the tumor suppressive role of GAS5 in HCC, including enhancing radiosensitivity, inhibiting invasion, and poor prognosis associated with its downregulation^38–40^. Collectively, our findings suggest a complex role for GAS5 in HCC, potentially playing a part in tumor initiation but also exerting a protective effect against disease progression.

Our study has some limitations. First, the study has inherent limitations related to patient demographics. The study population’s mean age of 63 years and predominantly male composition (80%) align with the typical HCC patient profile^41,42^. However, these characteristics might influence the model’s generalizability to populations with varying age and gender distributions. Another limitation is the relatively small sample size. While our findings provide valuable insights, a larger cohort could strengthen the generalizability of the results. We focused only on analyzing circulating lncRNA levels in plasma which is suitable for screening purposes. However, integrating this data with tissue expression levels of the same lncRNAs would have offered a more comprehensive perspective. This combined approach could have provided valuable validation for our findings, offering a deeper understanding of the role of these lncRNAs in HCC. Finally, it is important to emphasis that, although the performance of the models approaches 100% ,which is very promising, the model needs to be validated on different data sets before stepping forward to clinical application to minimize the effect of sample size and variability between data sets on the results.

### Conclusions and recommendations

Our study shows that lncRNAs offer moderate diagnostic value for HCC. However, the implementation of a machine learning model that incorporates lncRNAs with standard laboratory data significantly improves their diagnostic utility. This model can be readily translated into a user-friendly interface, such as a website or mobile application, facilitating convenient use by healthcare professionals. The simplicity of the model, coupled with the relative speed and affordability of the underlying laboratory tests, positions it as a promising tool for screening on a large-scale.

Future research directions include evaluating the model’s robustness and prognostic prediction capabilities on a larger patient cohort. Additionally, investigation into a broader panel of lncRNAs holds promise for further refinement and optimization of the model. Moreover, investigating the model’s ability to differentiate HCC from other benign liver diseases presents a promising avenue for future research.

## Electronic supplementary material

Below is the link to the electronic supplementary material.

Supplementary Material 1

Supplementary Material 2

## Data Availability

The datasets used and/or analyzed during the current study are available from the corresponding author on reasonable request.

## Declarations

### Study approval statement

The protocol was approved by the ethical committee of Shefaa Al Orman Oncology Hospital.

## References

[1] Forner, A., Reig, M., Bruix, J. & Hepatocellular carcinoma *Lancet.* 391, (10127):1301–1314 doi: (2018). 10.1016/S0140-6736(18)30010-2 10.1016/S0140-6736(18)30010-2 29307467

[2] Jemal, A. et al. Global cancer statistics. *Cancer J. Clin.* **61** (2), 69–90. 10.3322/caac.20107 (2011). 10.3322/caac.20107 21296855

[3] Villanueva, A. & Hepatocellular Carcinoma *N Engl. J. Med.* **380**(15), 1450–1462 doi:10.1056/NEJMra1713263 (2019). 30970190 10.1056/NEJMra1713263

[4] Rashed, W. M., Kandeil, M., Mahmoud, M. & Ezzat, S. Hepatocellular Carcinoma (HCC) in Egypt: a comprehensive overview. *J. Egypt. Natl. Canc Inst.* **32** (1), 5. 10.1186/s43046-020-0016-x (2020). 32372179 10.1186/s43046-020-0016-x PMC13325438

[5] El-Zayadi et al. Hepatocellular carcinoma in Egypt: a single center study over a decade. *World J. Gastroenterol.* **11** (33), 5193–5198. 10.3748/wjg.v11.i33.5193 (2005). 16127751 10.3748/wjg.v11.i33.5193 PMC4320394

[6] Ezzat, R., Eltabbakh, M. & Kassas, E. Unique situation of hepatocellular carcinoma in Egypt: a review of epidemiology and control measures. *W J. Gastrointest. Oncol.* **13** (12), 1919–1938. 10.4251/wjgo.v13.i12.1919 (2021). 10.4251/wjgo.v13.i12.1919 PMC8713321 35070033

[7] Das, M. Egypt launches 100 healthy days health-care campaign. *Lancet Oncol.* **24** (8), 845 (2023). 37454663 10.1016/S1470-2045(23)00338-8

[8] Tinkle, C. & Haas-Kogan, D. Hepatocellular carcinoma: natural history, current management, and emerging tools. *Biologics: Targets and Therapy*. 207 – 19 (2012). 10.2147/BTT.S23907 PMC3421475 22904613

[9] Poon, D. et al. Management of hepatocellular carcinoma in Asia: consensus statement from the Asian oncology Summit 2009. *Lancet Oncol.* **10** (11), 1111–1118 (2009). 19880065 10.1016/S1470-2045(09)70241-4

[10] Ge, X., Yao, Y., Li, J., Li, Z. & Han, X. Role of LncRNAs in the epithelial-mesenchymal transition in Hepatocellular Carcinoma. *Front. Oncol.* **11**, 690800. 10.3389/fonc.2021.690800 (2021). 34113574 10.3389/fonc.2021.690800 PMC8185227

[11] Khan, A. & Zhang, X. Function of the long noncoding RNAs in Hepatocellular Carcinoma: classification, Molecular mechanisms, and significant therapeutic potentials. *Bioeng. (Basel)*. **9** (8), 406 (2022). 10.3390/bioengineering9080406 PMC9405066 36004931

[12] Tümen, D. et al. Pathogenesis and current treatment strategies of hepatocellular carcinoma. *Biomedicines* **10** (12), 3202 (2022). 36551958 10.3390/biomedicines10123202 PMC9775527

[13] Yang, Y. et al. Recurrently deregulated lncRNAs in hepatocellular carcinoma. *Nat. Commun.* **8**, 14421. 10.1038/ncomms14421 (2017). 28194035 10.1038/ncomms14421 PMC5316832

[14] Huang, Z., Zhou, J. K., Peng, Y., He, W. & Huang, C. The role of long noncoding RNAs in hepatocellular carcinoma. *Mol. Cancer*. **19** (1), 77. 10.1186/s12943-020-01188-4 (2020). 32295598 10.1186/s12943-020-01188-4 PMC7161154

[15] Ge, W. J. et al. Long non-coding RNAs in hepatocellular carcinoma. *Pathol. Res. Pract.* **248**, 154604. 10.1016/j.prp.2023.154604 (2023). 37302276 10.1016/j.prp.2023.154604

[16] Pei, M. et al. LINC00152 promotes cell cycle progression in hepatocellular carcinoma via miR-193a/b-3p/CCND1 axis. *cell. Cycle*. **17** (8), 974–984. 10.1080/15384101.2018.1464834 (2018). 29895195 10.1080/15384101.2018.1464834 PMC6103663

[17] Qin, L. T. & et tal. Biological function of UCA1 in hepatocellular carcinoma and its clinical significance: investigation with in vitro and meta-analysis. *Pathol. Res. Pract.* **214** (9), 1260–1272. 10.1016/j.prp.2018.03.025 (2018). 30017333 10.1016/j.prp.2018.03.025

[18] Shah, M. & Sarkar, D. HCC-Related lncRNAs: roles and mechanisms. *Int. J. Mol. Sci.* **25** (1), 597. 10.3390/ijms25010597 (2024). 38203767 10.3390/ijms25010597 PMC10779127

[19] Zhang, W. Y. et al. Long noncoding RNA Gas5 induces cell apoptosis and inhibits tumor growth via activating the CHOP-dependent endoplasmic reticulum stress pathway in human hepatoblastoma HepG2 cells. *J Cell Biochem*. 123(2), 231–247 doi: (2022). 10.1002/jcb.30159. Epub 2021 Oct 11. PMID: 34636091. 10.1002/jcb.30159 34636091

[20] Abdelmoety, A.A. et al. The role of UCA1 and WRAP53 in diagnosis of hepatocellular carcinoma: a single-center case-control study. *Clin. Exp. Hepatol.* **9**(2) 129 – 37 (2023). 10.5114/ceh.2023.127569 PMC10369654 37502440

[21] Abdelrahman, E. et al. Serum long intergenic non-coding ribonucleic acid LINC00152 as a potential predictor of hepatocellular carcinoma in Egyptian patients. *Afro-Egyptian J. Infect. Endemic Dis.* **10** (3), 264–270 (2020).

[22] Li et al. Sun, B. HULC and Linc00152 act as novel biomarkers in predicting diagnosis of hepatocellular carcinoma. *Cell. Phys. Biochem.* **37** (2), 687–696 (2015). 10.1159/000430387 26356260

[23] Kim, S. S. & et tal. Serum small extracellular vesicle-derived LINC00853 as a novel diagnostic marker for early hepatocellular carcinoma. *Mol. Oncol.* **14** (10), 2646–2659 (2024). 10.1002/1878-0261.12745 PMC7530776 32525601

[24] Kamel, M. M. et al. Investigation of long noncoding RNAs expression profile as potential serum biomarkers in patients with hepatocellular carcinoma. *Transl Res.* **168**, 134–145 (2016). 26551349 10.1016/j.trsl.2015.10.002

[25] Yuan, D. et al. Long non-coding RNAs: potential biomarkers and targets for Hepatocellular Carcinoma Therapy and diagnosis. *Int. J. Biol. Sci.* **17** (1), 220–235. 10.7150/ijbs.50730 (2012). 10.7150/ijbs.50730 PMC7757045 33390845

[26] Shi, T., Morishita, A., Kobara, H. & Masaki, T. The role of long non-coding RNA and microRNA networks in hepatocellular carcinoma and its tumor microenvironment. *Intl J. Mole Sci.* **22** (19), 10630 (2021). 10.3390/ijms221910630 PMC8508708 34638971

[27] Pfaffl, M. W. Relative quantification. In: (eds T & Dorak) Real Time PCR BIOS Advanced Methods. New York, NY: Taylor & Francis. 63–82 (2006).

[28] Jin, Y. et al. Comprehensive analysis of transcriptome profiles in hepatocellular carcinoma. *J. Trans. Med.* **17**, 273. 10.1186/s12967-019-2025-x (2019). 10.1186/s12967-019-2025-x PMC6701074 31429776

[29] Sato, M. et al. Machine-learning Approach for the development of a Novel Predictive Model for the diagnosis of Hepatocellular Carcinoma. *Sci. Rep.* **9** (1), 7704 10.1038/s41598-019-44022-8(2019). 31147560 10.1038/s41598-019-44022-8 PMC6543030

[30] Angelis, I. & Exarchos, T. Hepatocellular Carcinoma Detection Using Machine Learning Techniques. *Adv. Exp. Med. Biol.* **1338**, 21–29. 10.1007/978-3-030-78775-2_4 (2021). 34973006 10.1007/978-3-030-78775-2_4

[31] Wong, G. L. et al. Novel machine learning models outperform risk scores in predicting hepatocellular carcinoma in patients with chronic viral hepatitis. *JHEP Rep.* **4** (3), 100441. 10.1016/j.jhepr.2022.100441 (2022). 35198928 10.1016/j.jhepr.2022.100441 PMC8844233

[32] Chen, S. et al. Using quasispecies patterns of Hepatitis B Virus to Predict Hepatocellular Carcinoma with Deep sequencing and machine learning. *J. Infect. Dis.* **223** (11), 1887–1896. 10.1093/infdis/jiaa647 (2021). 33049037 10.1093/infdis/jiaa647

[33] Tao, K. et al. Machine learning-based genome-wide interrogation of somatic copy number aberrations in circulating tumor DNA for early detection of hepatocellular carcinoma. *eBioMedicine* **56**, 102811. 10.1016/j.ebiom.2020.102811 (2020). 32512514 10.1016/j.ebiom.2020.102811 PMC7276513

[34] Li, S. et al. Long non-coding RNA LINC00152 in cancer: roles, mechanisms, and chemotherapy and radiotherapy resistance. *Front. Oncol.* **12**, 960193 10.3389/fonc.2022.960193 (2022). 36033524 10.3389/fonc.2022.960193 PMC9399773

[35] Wang, B., Yang, S. & Zhao, W. L. Non-coding RNA NRAD1 and LINC00152 are highly expressed and associated with prognosis in patients with hepatocellular carcinoma. *Onco Targets Ther.* **13**, 10409–10416. 10.2147/OTT.S251231 (2020). 33116620 10.2147/OTT.S251231 PMC7569076

[36] Deng, X. et al. Linc00152 promotes cancer progression in hepatitis B virus-associated hepatocellular carcinoma. *Biomed. Pharmacother*. **90**, 100–108. 10.1016/j.biopha.2017.03.031 (2017). 28343069 10.1016/j.biopha.2017.03.031

[37] Tian, Q. et al. CYTOR promotes cell proliferation and tumor growth via miR-125b/SEMA4C axis in hepatocellular carcinoma. *Oncol. Lett.* **22** (5), 796. 10.3892/ol.2021.13057 (2021). 34584571 10.3892/ol.2021.13057 PMC8461761

[38] Yang, L. & Jiang, J. GAS5 regulates RECK expression and inhibits Invasion potential of HCC cells by sponging miR-135b. *Biomed. Res. Int.* 20192973289. 10.1155/2019/2973289 (2019). 10.1155/2019/2973289 PMC6348854 30733959

[39] Chang, L. et al. Decreased expression of long non-coding RNA GAS5 indicates a poor prognosis and promotes cell proliferation and invasion in hepatocellular carcinoma by regulating vimentin. *Mol. Med. Rep.* **13** (2), 1541–1550. 10.3892/mmr.2015.4716 (2016). 26707238 10.3892/mmr.2015.4716 PMC4732840

[40] Yu, C., Liang, Y., Jin, Y. & Li, Q. LncRNA GAS5 enhances radiosensitivity of hepatocellular carcinoma and restricts tumor growth and metastasis by miR-144-5p/ATF2. *Am. J. Transl Res.* **13** (9), 10896–10907 (2021). 34650771 PMC8506991

[41] Ali, A. A., Gamal, S. E., Anwar, R., Elzahaf, E. & Eskandere, D. Assessment of clinico-epidemiological profile of hepatocellular carcinoma in the last two decades. *Egyp J. Inter Med.* **35** (1), 18 (2023).

[42] El-Serag, H. B. Hepatocellular carcinoma: recent trends in the United States. *Gastroenterology* **127** (5), 27–34 (2004). 10.1053/j.gastro.2004.09.013 15508094

