# Supplementary material for: Analysis of four long non-coding RNAs for hepatocellular carcinoma screening and prognosis by the aid of machine learning techniques
Source: Sci Rep. 2024 Nov 28;14:29582. doi: 10.1038/s41598-024-80926-w (PMC11604705; doi:10.1038/s41598-024-80926-w)
Supplement: Supplementary file 1 — Supplementary Material 1 [file 41598_2024_80926_MOESM1_ESM.docx]

Table 1: comparison between the ROC curve of long non-coding RNA

| Pair | LN0052, LN00853 | LN0052, UCA1 | LN0052, GAS5 | LN00853, UCA1 | LN00853, GAS5 | UCA1, GAS5 |
| --- | --- | --- | --- | --- | --- | --- |
| Area Difference | 0.01 | -0.03 | -0.03 | -0.03 | -0.04 | -0.01 |
| Standard Error | 0.07 | 0.07 | 0.08 | 0.07 | 0.07 | 0.07 |
| 95% Confidence Interval | -0.1292 to 0.1442 | -0.1672 to 0.1166 | -0.1962 to 0.1330 | -0.1686 to 0.1030 | -0.1850 to 0.1069 | -0.1476 to 0.1350 |
| P Value | 0.91 | 0.73 | 0.71 | 0.64 | 0.60 | 0.93 |

Table 2: comparison between the ROC curve of AST, ALT and AFP

| Pair | AST, ALT | AST, AFP | ALT, AFP |
| --- | --- | --- | --- |
| Area Difference | 0.08 | 0.25 | 0.17 |
| Standard Error | 0.04 | 0.06 | 0.07 |
| 95% Confidence Interval | 0.009152 to 0.1547 | 0.1406 to 0.3682 | 0.04448 to 0.3004 |
| P Value | 0.03 | < 0.0001 | 0.01 |

The comparison between the ROC curve area showed that AST was the most superior on ALT and AFP, as well ALT was more superior on AFP, p<0.05. However, there was insignificant difference between all long non-coding RNA markers, p>0.05.

Table 3: Cox regression analysis of lncRNAs expression in predicting mortality of HCC.

| **Covariate** | CE | HR | 95% CI-L | 95% CI-U | P Value |
| --- | --- | --- | --- | --- | --- |
| LN0052 (< 2.78) | 0.10 | 1.11 | 0.53 | 2.33 | 0.79 |
| LN00853 (<1.55) | -0.15 | 0.86 | 0.46 | 1.60 | 0.63 |
| UCA1 (<1.99) | -0.23 | 0.79 | 0.34 | 1.84 | 0.59 |
| GAS5 (<0.68) | 0.09 | 1.09 | 0.49 | 2.44 | 0.83 |
| Gender-Female | -0.61 | 0.54 | 0.24 | 1.24 | 0.15 |
| Age | -0.01 | 0.99 | 0.95 | 1.02 | 0.38 |
